# Passive Tracking of Multiple Underwater Targets in Incomplete Detection and Clutter Environment

**DOI:** 10.3390/e23081082

**Published:** 2021-08-20

**Authors:** Xiaohua Li, Bo Lu, Wasiq Ali, Haiyan Jin

**Affiliations:** 1 School of Computer Science and Engineering, Xi’an University of Technology, Xi’an 710048, China; LB18098047330@163.com (B.L.); jinhaiyan@xaut.edu.cn (H.J.); 2 Shaanxi Key Laboratory for Network Computing and Security Technology, Xi’an 710048, China; 3 School of Marine Science and Technology, Northwestern Polytechnical University, Xi’an 710072, China; wasiqali@mail.nwpu.edu.cn

**Keywords:** dense clutter, data association uncertainty, passive target tracking, Doppler and bearing, Bayesian filter, underwater, multiple targets, tracking, cardinalized probability hypothesis density

## Abstract

A major advantage of the use of passive sonar in the tracking multiple underwater targets is that they can be kept covert, which reduces the risk of being attacked. However, the nonlinearity of the passive Doppler and bearing measurements, the range unobservability problem, and the complexity of data association between measurements and targets make the problem of underwater passive multiple target tracking challenging. To deal with these problems, the cardinalized probability hypothesis density (CPHD) recursion, which is based on Bayesian information theory, is developed to handle the data association uncertainty, and to acquire existing targets’ numbers and states (e.g., position and velocity). The key idea of the CPHD recursion is to simultaneously estimate the targets’ intensity and the probability distribution of the number of targets. The CPHD recursion is the first moment approximation of the Bayesian multiple targets filter, which avoids the data association procedure between the targets and measurements including clutter. The Bayesian-filter-based extended Kalman filter (EKF) is applied to deal with the nonlinear bearing and Doppler measurements. The experimental results show that the EKF-based CPHD recursion works well in the underwater passive multiple target tracking system in cluttered and noisy environments.

## 1. Introduction

The tracking of multiple underwater targets using passive sonar (e.g., bearings-only multiple-target tracking; bearing and Doppler multiple target tracking) is receiving a great deal of attention in practical defense and civil applications [1,2,3]. Passive multiple-target tracking aims to obtain the number of expected targets in the tracking space, as well as the states of the targets, from passive measurements such as bearing, bearing rate, Doppler, Doppler rate, and the time arrival deference. The greatest advantage of passive multiple target tracking systems is that passive sonar emits no signal, and thus can be kept covert when conducting the passive measurements such as bearings measurements and Doppler frequency measurements, avoiding the risk of being tracked [4]. In addition, passive sonar tracking systems are simple and small in terms of configuration, and have low maintenance costs.

The main challenges associated with passive multiple underwater target tracking are that the passively obtained information is highly nonlinear [5,6,7], the targets’ range may be unobservable, and the data association uncertainty between passive measurements and targets is complicated.

The range unobservability for bearings-only target tracking means that the passive sonar sensors cannot obtain accurate range information about the targets [8,9,10,11]. The general methods to avoid the target range unobservability include the use of more than one stationary or maneuvering passive observation station, or the use of a single maneuvering passive sensor. In this paper, we introduce Doppler frequency measurement to avoid the range unobservability problem. By using the information obtained from bearing and Doppler measurements, targets’ range state is observable even if the passive sonar is static [8].

In order to deal with nonlinear data such as bearings and Doppler, the extended Kalman filter (EKF) is used [11]. The EKF is a nonlinear and non-Gaussian Bayesian filtering algorithm that locally linearizes targets’ state and measurement equations using the first part of the Taylor expansion of the nonlinear transformations around the expected predicted target state. The results may be unsatisfactory when the tracking system is highly nonlinear and non-Gaussian. Another nonlinear filter method is the unscented Kalman filter (UKF) [12,13], which is an unscented transform approximation to the tracking system function based on deterministic sampling instead of the linear approximation used in the EKF. The UKF has better tracking performance than the EKF to some extent, but its computational cost is larger than that of the EKF. The other typical nonlinear method is the particle filter (PF) [14,15], which forms the Monte Carlo approximation to the solution of the Bayesian filter, and uses a set of particle samples to approximate the targets’ distribution according to the targets’ probability density. The PF is used in cases where the tracking model is highly nonlinear or non-Gaussian. One disadvantage of the PF is its high computational cost. In this paper we apply the EKF to deal with the nonlinear bearing and Doppler measurements.

Another difficulty associated with passive multiple underwater target tracking is the data association uncertainties between targets and measurements [16]. The most simple data association algorithm is the global nearest neighbor (GNN) method, which attempts to obtain the most likely hypothesis between targets and measurements. The other traditional data association approach to solve the uncertainties problem between targets and measurements is the joint probabilistic data association (JPDA) method [17]; its computing cost is exponentially increased with the number of measurements, interferences, and targets. In addition, the multiple hypothesis tracking (MHT) method [18], the probabilistic multiple hypothesis tracking (PMHT) method [19], and their improved algorithms are also used to handle data association uncertainties between targets and measurements. The main disadvantage of these traditional data association multiple target tracking algorithms (GNN, JPDA, MHT, PMHT) is that they cannot deal with the time-variant and space-variant tracking problem. Recently, the random finite set (RFS) has emerged as a promising method which does not rely on the data association [20,21,22,23,24,25,26]. RFS-based methods treat multiple target states as the state finite set. In the same way, they treat measurement data as the measurement finite set.

One of the most popular RFS methods is probability hypothesis density (PHD) recursion, which can estimate multiple targets’ states as well as the number of targets through propagating the targets’ posterior intensity RFS to the Bayesian multiple targets filter [25]. Another popular method is cardinalized probability hypothesis density (CPHD) recursion [27], which assumes the higher order on the number of targets without considering data associations between targets and measurements, and jointly propagates the targets’ posterior probability and the number of targets’ (i.e., the cardinality) density distribution [27]. PHD and the CPHD both make all the measurements as a measurement set and all the targets as a target set.

Information entropy theories are also used to estimate single target states and multiple targets states. The fuzzy c-means clustering method based on maximum information entropy combined with PDA is proposed in [28], which uses a value optimized by the maximum information entropy to represent the measurement-to-target association probability. The multiple target tracking problem has also been solved by the maximum entropy intuitionistic fuzzy data association algorithm [29], cross entropy [30], maximum-fuzzy-entropy-based Gaussian clustering algorithm [31], entropy distribution and game theory based on the random finite set probability hypothesis density (PHD) method [32], maximum entropy fuzzy based on the fire-fly and PF [33], and the distributed cross-entropy-based δ-generalized labeled multi-Bernoulli (δ-GLMB) filter [34].

In this paper we investigate the tracking performance of CPHD recursion in passive underwater multiple target tracking in the two-dimensional state space under a cluttered environment, using bearing and Doppler measurements. We also consider the case of a single stationary passive sonar observer. In order to improve the tracking performance, the EKF is used to deal with the nonlinear bearing and Doppler measurements with independent Gaussian white noise. In order to avoid the range unobservability problem, in this paper we introduce nonlinear Doppler measurement. The CPHD, which can jointly estimate the state and number of targets, is used to handle the targets-to-measurements data association uncertainty in the cluttered environment.

The remainder of the paper is organized as follows. The passive multiple underwater target tracking system model using Doppler and bearing measurements is given in Section 2. Section 3 develops the recursive CPHD suitable for multiple targets tracking under a dense clutter environment. The simulation results are given in Section 4. Lastly, a summary is given in Section 5.

## 2. Signal Model and Problem Formulation

### 2.1. System Model

We assume that the number of targets is variable and unknown during the entire tracking procedure in two-dimensional Cartesian space. In this paper, we consider the dynamic nearly constant turn (CT) system model for all the targets with Gaussian process noise [35]. Some maneuvering target tracking problems are discussed in [36,37].

The state for target m at time t is

(1) $${\tilde{x}}_{t,m}={\left(x_{t,m},\;{\dot{x}}_{t,m},\;y_{t,m},\;{\dot{y}}_{t,m},\;w_{t,m}\right)}^{T}$$

in which $w_{t,m}$ is the target’s turn rate, and

(2) $$x_{t,m}={\left(x_{t,m},\;{\dot{x}}_{t,m},\;y_{t,m},\;{\dot{y}}_{t,m}\right)}^{T}$$

is the target’s position and velocity, where the target’s position is $\left(x_{t,m},\;y_{t,m}\right)$, the target’s velocity is $\left({\dot{x}}_{t,m},\;{\dot{y}}_{t,m}\right)$, and t=1,2,⋯,T is the time index.

**Figure 1:**
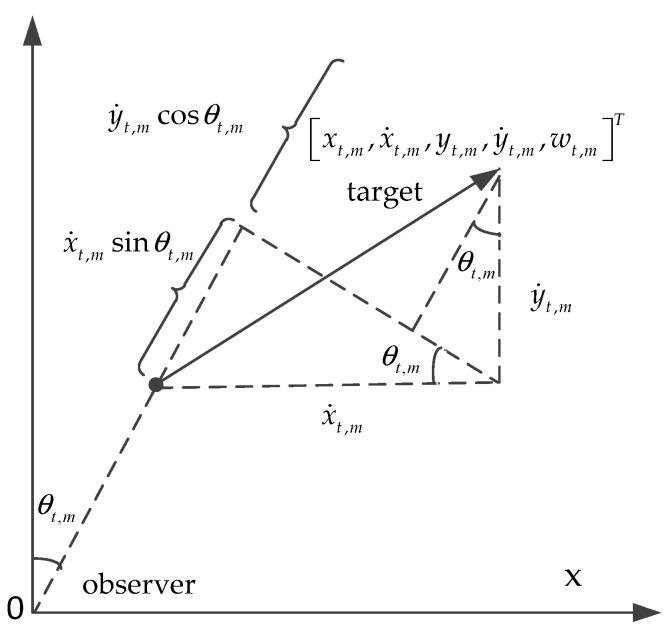
An overview of the bearing and Doppler target tracking (passive sensor) geometry.

The targets to single stationary observer station (passive sensor) tracking scenario using bearing and Doppler measurements information is depicted in Figure 1.

The target transition model with process noise is given by

(3) $$x_{t,m}=F_{t,m}x_{t,m}+w_{t,m}$$

where $w_{t,m}$ is the system process noise.

For the nearly CT target tracking model, $F_{t,m}$ is a system state transition matrix which is given by

(4) $$F_{t,m}=\left[\begin{array}{cccc} 1 & \frac{\sin w\Delta T}{w\Delta T} & 0 & -\frac{1-\cos w\Delta T}{w\Delta T}\\ 0 & \cos w\Delta T & 0 & -\sin w\Delta T\\ 0 & \frac{1-\cos w\Delta T}{w\Delta T} & 1 & \frac{\sin w\Delta T}{w\Delta T}\\ 0 & \sin w\Delta T & 0 & \cos w\Delta T \end{array}\right]$$

in which ΔT is the sampling interval.

This paper supposes the tracking process noise $w_{t,m}$ is Gaussian white noise with covariance

(5) $$Q_{t,m}=\delta_{p}^{2}\left[\begin{array}{cccc} \frac{\Delta T^{4}}{4} & \frac{\Delta T^{3}}{2} & 0 & 0\\ \frac{\Delta T^{3}}{2} & \Delta T^{2} & 0 & 0\\ 0 & 0 & \frac{\Delta T^{4}}{4} & \frac{\Delta T^{3}}{2}\\ 0 & 0 & \frac{\Delta T^{3}}{2} & \Delta T^{2} \end{array}\right]$$

where $\delta_{p}^{2}$ is the acceleration noise intensity.

### 2.2. Measurement Model

In this paper, we assume a single stationary passive sensor located at the original coordinates. We assume the single stationary passive sensor can only measure the targets’ bearings and Doppler information, which are both nonlinear with respect to the passive sensor and targets’ state. 

As seen in Figure 1, for target m, the measurement can be modeled as

(6) $$z_{t,m}=h_{t,m}+u_{t,m}=h_{t,m}\left(x_{t,m},u_{t,m}\right)$$

where $h_{t,m}$ is the bearing and Doppler measurement function and $u_{t,m}$ is measurement noise.

We assume the bearing and Doppler measurement noise $u_{t,m}$ is Gaussian white noise with covariance

(7) $$R_{t,m}=\left[\begin{array}{c} u_{\theta ,t,m}\\ u_{D,t,m} \end{array}\right]$$

where $u_{\theta ,t,m}$ is bearing noise covariance and $u_{D,t,m}$ is Doppler noise covariance.

This paper assumes that the tracking process noise and measurement noise are independent.

The bearing and Doppler measurement information function $h_{t,m}$ is as follows:

(8) $$h_{t,m}=\left[\begin{array}{c} \arctan\frac{x_{t,m}}{y_{t,m}}\\ \left[1-\frac{{\dot{x}}_{t,m}\sin\theta_{t,m}+{\dot{y}}_{t,m}\cos\theta_{t,m}}{c}\right]f_{0} \end{array}\right]$$

where $\theta_{t,m}$ is the bearing of target m, c is the transmit speed of sound, and $f_{0}$ is the underwater targets’ radiant frequency.

## 3. CPHD Recursion Based on Bayesian Theory

This section first presents the multiple target tracking situation under the RFS framework. Then, the nonlinear EKF-based CPHD recursion for bearing and Doppler multiple target tracking is developed.

### 3.1. RFS Formulation of Multiple Target Filtering

In time t, suppose that the targets’ states are $x_{t,1},x_{t,2},\cdots x_{t,M\left(t\right)}\in 𝒳$ in which M(t) is the number of targets in time t. At the next time, some existing targets may be disappear, and some fresh targets may enter into the tracking space. For the bearing and Doppler measurements obtained by the passive sensor, N(t) is the number of measurements in time t. The bearing and Doppler measurements at time t are $z_{t,1},\cdots z_{t,N\left(t\right)}\in 𝒵$. The purpose of multiple target tracking is to jointly estimate the number of targets in the surveillance volume as well as their states from measurements with noise and clutter. 

The collection of target states at time t can be modeled as a target RFS,

(9) $$X_{t}=\left\{x_{t,1},\cdots x_{t,M\left(t\right)}\right\}\in ℱ\left(𝒳\right)$$

The collection of bearing and Doppler measurements at time t can be modeled as a measurement random finite set,

(10) $$Z_{t}=\left\{z_{t,1},\cdots z_{t,N\left(t\right)}\right\}\in ℱ\left(𝒵\right)$$

in which ℱ(𝒳) is the collection of all targets’ random finite subsets of 𝒳, and ℱ(𝒵) is the collection of all measurement random finite subsets of 𝒵.

Both the targets’ finite set $X_{t}$ and the bearing and Doppler measurement finite set $Z_{t}$ are out of order. 

The key of RFS is to treat $X_{t}$ as the multiple targets’ state, and to treat $Z_{t}$ as the multiple target observation. 

In this paper we model the multiple targets’ dynamics at time t as

(11) $$X_{t}=\left[\underset{x_{t-1}\in X_{t-1}}{\cup }S_{t|t-1}\left(x_{t-1}\right)\right]\cup \Gamma_{t}$$

where $X_{t-1}$ is the multiple targets’ state at time t−1, which, including the existing targets at time t−1 and the fresh targets which appear at time t, $S_{t|t-1}\left(x_{t-1}\right)$ is the existing target RFS at time t, $\Gamma_{t}$ is the RFS of spontaneous births at time t.

The multiple targets’ bearing and Doppler measurements, including clutter at time t, is

(12) $$Z_{t}=\left[\underset{x\in X_{t}}{\cup }\Theta_{t}\left(x\right)\right]\cup K_{t}$$

where $\Theta_{t}\left(x\right)$ is the bearing and Doppler measurement from target x, and $K_{t}$ is the RFS of clutter measurements.

Let $f_{t|t-1}\left(\cdot |\cdot \right)$ be the multiple targets’ transition density. Similarly, let $h_{t|t-1}\left(\cdot |\cdot \right)$ be the multiple targets’ likelihood. The posterior density of the multiple targets’ states is denoted by $p_{t}\left(\cdot |Z_{1:t}\right)$ The RFS formulation of multiple target Bayesian filtering propagates the targets’ posterior probability using the following equations:

(13) $$p_{t|t-1}\left(X_{t}|Z_{1:t-1}\right)=\int f_{t|t-1}\left(X_{t}|X\right)p_{t-1|t-1}\left(X|Z_{1:t-1}\right)\delta X$$

(14) $$p_{t}\left(X_{t}|Z_{1:t}\right)=\frac{h_{t}\left(Z_{t}|X_{t}\right)p_{t|t-1}\left(X_{t}|Z_{1:t-1}\right)}{\int h_{t}\left(Z_{t}|X\right)p_{t|t-1}\left(X|Z_{1:t-1}\right)\delta X}$$

where the integrals in Equations (13) and (14) are set integrals.

In practical situations, the Bayesian recursion in Equations (13) and (14) are intractable due to the multiple integrations. Suboptimal algorithms like PHD and CPHD are used to solve the integrals problem.

### 3.2. CPHD Recursion

The key idea of the CPHD Bayesian filter is to jointly propagate the multiple targets’ intensity equation and the cardinality probability distribution [38,39].

In order to evaluate the CPHD, we assume that:

- Each expected target produces bearing and Doppler measurements independently from one another;
- The targets’ birth RFS is independent of the surviving target RFS;
- The clutter’s RFS is independent from the true targets’ measurement RFS;
- The prior and predicted multiple targets’ RFSs are both independent and identically distributed processes.

In order to evaluate the CPHD algorithm, we denote the following:

The binomial coefficient is given by $C_{j}^{l}=\frac{l!}{j!\left(l-j\right)!}$, the permutation coefficient is given by $P_{j}^{n}=\frac{n!}{\left(n-j\right)!}$, the inner product between α and β is 〈α,β〉=∫α(x)β(x)dx, or $\langle \alpha ,\beta \rangle =\sum_{l=0}^{\infty }\alpha \left(l\right)\beta \left(l\right)$, the elementary symmetric function of j for a FST Z is $e_{j}\left(Z\right)=\sum_{S\subseteq Z,\left|S\right|=j}\left(\prod_{\zeta \in S}\zeta \right)$, and $e_{0}\left(Z\right)=1$.

Let $v_{t|t-1}\left(x\right)$ denote the predicted targets’ intensity at time t, and $p_{t|t-1}\left(n\right)$ denote the predicted targets’ cardinality at time t. Similarly, let $v_{t|t}\left(x\right)$ denote the targets’ posterior intensity and $p_{t|t}\left(n\right)$ denote the targets’ cardinality function at time t. 

Suppose one knows that the target posterior intensity is $v_{t-1}\left(x\right)$ and the target posterior cardinality is $p_{t-1}\left(n\right)$; then, the predicted target intensity $v_{t|t-1}\left(x\right)$ and predicted target cardinality $p_{t|t-1}\left(n\right)$ are:

(15) $$v_{t|t-1}\left(x\right)=\int p_{S,t|t-1}\left(\zeta \right)f_{t|t-1}\left(x|\zeta \right)v_{t-1}\left(\zeta \right)d\zeta +\gamma_{t}\left(x\right)$$

(16) $$p_{t|t-1}\left(n\right)=\sum_{j=0}^{n}p_{\Gamma ,t}\left(n-j\right)\Pi_{t|t-1}\left[v_{t-1},p_{t-1}\right]\left(j\right)$$

where

(17) $$\Pi_{t|t-1}\left[v_{t-1},p_{t-1}\right]\left(j\right)=\sum_{l=j}^{\infty }C_{j}^{l}\frac{{\langle p_{S,t},v\rangle }^{j}{\langle 1-p_{S,t},v\rangle }^{l-j}}{{\langle 1,v\rangle }^{l}}$$

and where $p_{S,t|t-1}\left(\zeta \right)$ is the probability of target existence, $\gamma_{t}\left(\cdot \right)$ is target birth intensity, and $p_{\Gamma ,t}\left(\cdot \right)$ is target birth cardinality.

Assume that the predicted intensity $v_{t|t-1}\left(x\right)$ and predicted target cardinality $p_{t|t-1}\left(n\right)$ are known. Then, the updated intensity $v_{t}\left(x\right)$ and updated cardinality distribution $p_{t}\left(n\right)$ at time t are given by

(18) $$v_{t}\left(x\right)=\frac{\langle {ϒ}_{t}^{1}\left[v_{t|t-1}^{},Z_{t}^{}\right],p_{t|t-1}^{}\rangle }{\langle {ϒ}_{t}^{0}\left[v_{t|t-1}^{},Z_{t}^{}\right],p_{t|t-1}^{}\rangle }\left[1-p_{D,t}\left(x\right)\right]v_{t|t-1}\left(x\right)+\sum_{z\in Z_{t}}\frac{\langle {ϒ}_{t}^{1}\left[v_{t|t-1},Z_{t}\backslash \left\{z\right\}\right],p_{t|t-1}\rangle }{\langle {ϒ}_{t}^{0}\left[v_{t|t-1},Z_{t}\right],p_{t|t-1}\rangle }\psi_{t,z}\left(x\right)v_{t|t-1}\left(x\right)$$

(19) $$p_{t}\left(n\right)=\frac{{ϒ}_{t}^{0}\left[v_{t|t-1},Z_{t}\right]\left(n\right)p_{t|t-1}\left(n\right)}{\langle {ϒ}_{t}^{0}\left[v_{t|t-1},Z_{t}\right],p_{t|t-1}\rangle }$$

where

(20) $${ϒ}_{t}^{u}\left[v,Z\right]\left(n\right)=\sum_{j=0}^{\min\left(\left|Z\right|,n\right)}\left(\left|Z\right|-j\right)p_{K,t}\left(\left|Z\right|-j\right)p_{j+u}^{n}\frac{{\langle 1-p_{D,t},v\rangle }^{n-\left(j+u\right)}}{{\langle 1,v\rangle }^{n}}e_{j}\left(\Xi_{t}\left(v,Z\right)\right)$$

(21) $$\psi_{t,z}^{}\left(x\right)=\frac{\langle 1,\kappa_{t}\rangle }{\kappa_{t}\left(z\right)}h_{t}\left(z|x\right)p_{D,t}^{}\left(x\right)$$

(22) $$\Xi_{t}\left(v,Z\right)=\left\{\langle v,\psi_{t,z}\rangle :z\in Z\right\}$$

and where $p_{D,t}^{}\left(x\right)$ is the target detection probability, $\kappa_{t}\left(\cdot \right)$ is the clutter intensity at time t, and $p_{K,t}^{}\left(\cdot \right)$ is the clutter cardinality distribution. 

Equation (19) is a Bayesian update, ${ϒ}_{t}^{0}\left[v_{t|t-1}^{},Z_{t}^{}\right]\left(n\right)$ is the likelihood of the multiple target observation set $Z_{t}^{}$ given that there are n targets, and $\langle {ϒ}_{t}^{0}\left[v_{t|t-1}^{},Z_{t}^{}\right],p_{t|t-1}^{}\rangle$ is the normalizing constant.

### 3.3. EKF-Based CPHD Recursion

This subsection develops the EKF-based CPHD recursion to the linear Gaussian system model in (3) and the nonlinear Gaussian measurement model (bearing and Doppler measurement model) in (6). 

According to Equations (3) and (6), the target model $x_{t,m}=F_{t,m}x_{t,m}+w_{t,m}$ is a linear Gaussian model, while the bearing and Doppler measurement model $z_{t,m}=h_{t,m}\left(x_{t,m},v_{t,m}\right)$ is a nonlinear Gaussian model. Due to the nonlinearities of measurement function $h_{t,m}$, the targets’ posterior function is nonlinear and non-Gaussian. Analogous to EKF, the linear and Gaussian CPHD recursion is extended to the nonlinear model by linearizing the nonlinear bearing and Doppler measurement function $h_{t,m}$.

The linearity for the measurement function $h_{t,m}$ of EKF is given by

(23) $$H_{t,m}=\frac{\partial h_{t,m}\left(x_{m},0\right)}{\partial x_{m}}$$

Next, we will develop the linear Gaussian CPHD recursion, then extend it to the nonlinear Gaussian model of bearing and Doppler measurements.

For the linear Gaussian system and measurement model, each target and sensor measurements are presented by a linear Gaussian function,

(24) $$f_{t|t-1}(x|\zeta )=𝒩(x;F_{t|t-1}\zeta ,Q_{t})$$

(25) $$h_{t|t-1}(z|x)=𝒩(z;H_{t}x,R_{t})$$

where 𝒩(·;m,P) is a Gaussian distribution whose mean is m and covariance matrix is P, and the other parameters have been defined above. 

The survival probability $p_{S,t}\left(x_{t-1}\right)$ and detection probability $p_{D,t}\left(x\right)$ are state independent for the linear Gaussian model, that is

(26) $$p_{S,t}\left(x_{t-1}\right)=p_{S,t}$$

(27) $$p_{D,t}\left(x\right)=p_{D,t}$$

The target birth RFS intensity for a linear Gaussian system and measurement model is given as

(28) $$\gamma_{t}\left(x\right)=\sum_{i=1}^{J_{\gamma ,t}}w_{\gamma ,t}^{\left(i\right)}𝒩(x;m_{\gamma ,t}^{\left(i\right)},P_{\gamma ,t}^{\left(i\right)})$$

where $w_{\gamma ,t}^{\left(i\right)}$ is the target weight, $m_{\gamma ,t}^{\left(i\right)}$ is the target mean, and $P_{\gamma ,t}^{\left(i\right)}$ is the target covariance. All of these values represent the target birth intensity distribution.

Assume the targets’ posterior probability hypothesis density $v_{t-1}\left(x\right)$ and the targets’ posterior cardinality $p_{t-1}\left(n\right)$ for a linear Gaussian system, and the bearing and Doppler measurement model are given. The posterior probability hypothesis density $v_{t-1}\left(x\right)$ is as follows:

(29) $$v_{t-1}\left(x\right)=\sum_{i=1}^{J_{t-1}}w_{t-1}^{\left(i\right)}𝒩(x;m_{t-1}^{\left(i\right)},P_{t-1}^{\left(i\right)})$$

Then, under the condition of linear and Gaussian assumption, the predicted target probability $v_{t|t-1}\left(x\right)$ and the predicted target cardinality are also Gaussian mixture, given by

(30) $$v_{t|t-1}\left(x\right)=\gamma_{t}(x)+p_{S,t|t-1}\sum_{i=1}^{J_{t-1}}w_{t-1}^{\left(i\right)}𝒩(x;m_{S,t|t-1}^{\left(i\right)},P_{S,t|t-1}^{\left(i\right)})$$

(31) $$p_{t|t-1}\left(n\right)=\sum_{j=0}^{n}p_{\Gamma ,t}\left(n-j\right)\sum_{l=j}^{\infty }C_{j}^{l}p_{t-1}\left(l\right)p_{S,t|t-1}^{j}{\left(1-p_{S,t}^{}\right)}^{l-j}$$

where the birth random finite set intensity for target $\gamma_{t}\left(x\right)$ is given in (25), and

(32) $$m_{S,t|t-1}^{\left(i\right)}=F_{t|t-1}^{}m_{t-1}^{\left(i\right)}$$

(33) $$P_{S,t|t-1}^{\left(i\right)}=F_{t|t-1}^{}P_{t-1}^{\left(i\right)}F_{t|t-1}^{T}+Q_{t}$$

For the linear Gaussian system and measurement model, suppose that the predicted probability hypothesis density $v_{t|t-1}\left(x\right)$ and the predicted target cardinality $p_{t|t-1}\left(n\right)$ at time t are given, and the predicted probability hypothesis density $v_{t|t-1}\left(x\right)$ is a Gaussian mixture distribution. Suppose that the target posterior probability hypothesis density $v_{t}\left(x\right)$ is also Gaussian mixture at time t, and

(34) $$\begin{array}{l} v_{t}\left(x\right)=[1-p_{D,t}]\frac{\langle \Psi_{t}^{1}\left[w_{t|t-1}^{},Z_{t}^{}\right],p_{t|t-1}^{}\rangle }{\langle \Psi_{t}^{0}\left[w_{t|t-1}^{},Z_{t}^{}\right],p_{t|t-1}^{}\rangle }v_{t|t-1}^{}(x)\\ \;\;\;+p_{D,t}\sum_{z\in Z_{t}}^{}\sum_{i=1}^{J_{t|t-1}^{}}w_{t|t-1}^{\left(i\right)}\frac{\langle \Psi_{t}^{1}\left[w_{t|t-1}^{},Z_{t}^{}\backslash \{z\}\right],p_{t|t-1}^{}\rangle }{\langle \Psi_{t}^{0}\left[w_{t|t-1}^{},Z_{t}^{}\right],p_{t|t-1}^{}\rangle }\frac{q_{t}^{\left(i\right)}(z)𝒩(x;m_{t}^{\left(i\right)}(z),P_{t}^{\left(i\right)})}{\kappa_{t}\left(z\right)/\langle 1,\kappa_{t}\rangle } \end{array}$$

(35) $$p_{t}\left(n\right)=\frac{\Psi_{t}^{0}\left[w_{t|t-1}^{},Z_{t}^{}\right]\left(n\right)p_{t|t-1}^{}\left(n\right)}{\langle \Psi_{t}^{0}\left[w_{t|t-1}^{},Z_{t}^{}\right],p_{t|t-1}^{}\rangle }$$

in which

(36) $$\Psi_{t}^{u}\left[w,Z\right]\left(n\right)=\sum_{j=0}^{\min\left(\left|Z\right|,n\right)}\left(\left|Z\right|-j\right)p_{K,t}^{}\left(\left|Z\right|-j\right)p_{j+u}^{n}\frac{{\langle 1-p_{D,t},v\rangle }^{n-\left(j+u\right)}}{{\langle 1,w\rangle }^{j+u}}e_{j}\left(\Lambda_{t}\left(w,Z\right)\right)$$

(37) $$\Lambda_{t}\left(w,Z\right)=\left\{\frac{p_{D,t}^{}w^{T}q_{t}\left(z\right)}{\kappa_{t}\left(z\right)/\langle 1,\kappa_{t}\rangle }:z\in Z\right\}$$

(38) $$w_{t|t-1}={\left[w_{t|t-1}^{\left(1\right)},\cdots ,w_{t|t-1}^{\left(J_{t|t-1}\right)}\right]}^{T}$$

(39) $$q_{t}\left(z\right)={\left[q_{t}^{\left(1\right)}\left(z\right),\cdots ,q_{t}^{\left(J_{t|t-1}\right)}\left(z\right)\right]}^{T}$$

(40) $$q_{t}^{\left(i\right)}(z)=𝒩(z;\eta_{t|t-1}^{\left(i\right)},S_{t|t-1}^{\left(i\right)})$$

(41) $$\eta_{t|t-1}^{\left(i\right)}=H_{t}m_{t|t-1}^{\left(i\right)}$$

(42) $$S_{t}^{\left(i\right)}=H_{t}P_{t|t-1}^{\left(i\right)}H_{t}^{T}+R_{t}$$

(43) $$m_{t}^{\left(i\right)}\left(z\right)=m_{t|t-1}^{\left(i\right)}+K_{t}^{\left(i\right)}\left(z-\eta_{t|t-1}^{\left(i\right)}\right)$$

(44) $$P_{t}^{\left(i\right)}=\left[I-K_{t}^{\left(i\right)}H_{t}\right]P_{t|t-1}^{\left(i\right)}$$

(45) $$K_{t}^{\left(i\right)}=P_{t|t-1}^{\left(i\right)}H_{t}^{T}{\left[S_{t|t-1}^{\left(i\right)}\right]}^{-1}$$

For the linear target model and the nonlinear bearing and Doppler measurement model in this paper, the update step for the CPHD recursion is to approximate the nonlinear bearing and Doppler measurement model. That is, predicted targets are updated by using the first order of the Taylor extension when a nonlinear composition appeared [33]. That is, Equations (46) and (47) are used instead of Equations (35) and (36), and Equation (48) is used for the calculation of (46) and (47)

(46) $$\eta_{t|t-1}^{\left(i\right)}=h_{t}\left(m_{t|t-1}^{\left(i\right)},0\right)$$

(47) $$S_{t}^{\left(i\right)}=H_{t}^{\left(i\right)}P_{t|t-1}^{\left(i\right)}{\left[H_{t}^{\left(i\right)}\right]}^{T}+U_{t}^{\left(i\right)}R_{t}{\left[U_{t}^{\left(i\right)}\right]}^{T}$$

in which

(48) $$H_{t}^{\left(i\right)}={\frac{\partial h_{t}\left(x,0\right)}{\partial x}|}_{x=m_{t|t-1}^{\left(i\right)}},U_{t}^{\left(i\right)}={\frac{\partial h_{t}\left(m_{t|t-1}^{\left(i\right)},v\right)}{\partial v}|}_{v=0}$$

## 4. Simulations

This section presents a simulation of a nonlinear passive underwater multiple target tracking scenario in a cluttered environment using bearing and Doppler measurements to show the tracking performance of the EKF-based CPHD recursion. The multiple targets moved with the CT model with a varying turning rate in a space of [0, 3000] m × [200, 2500] m. The number of targets was time varying, and they appeared and disappeared at different times and positions. The maximum number of targets was 4.

**Figure 2:**
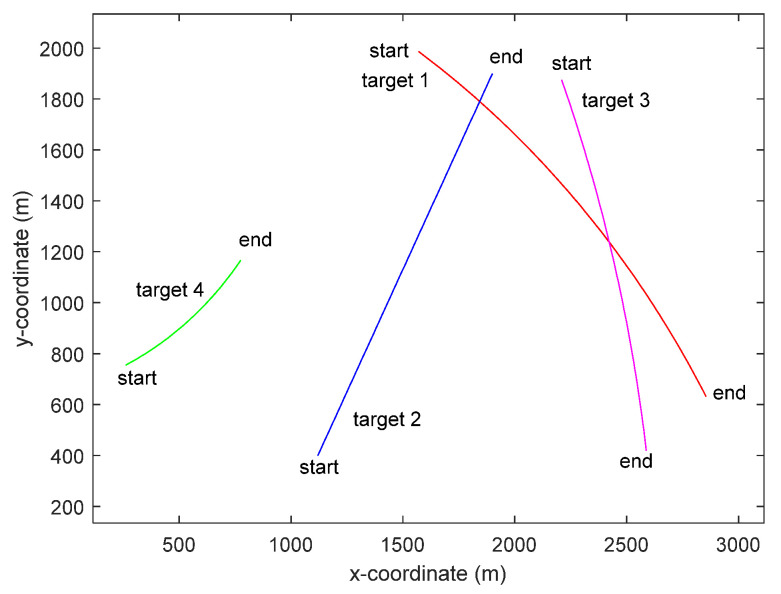
The true states for each target in the two-dimensional surveillance region.

**Figure 3:**
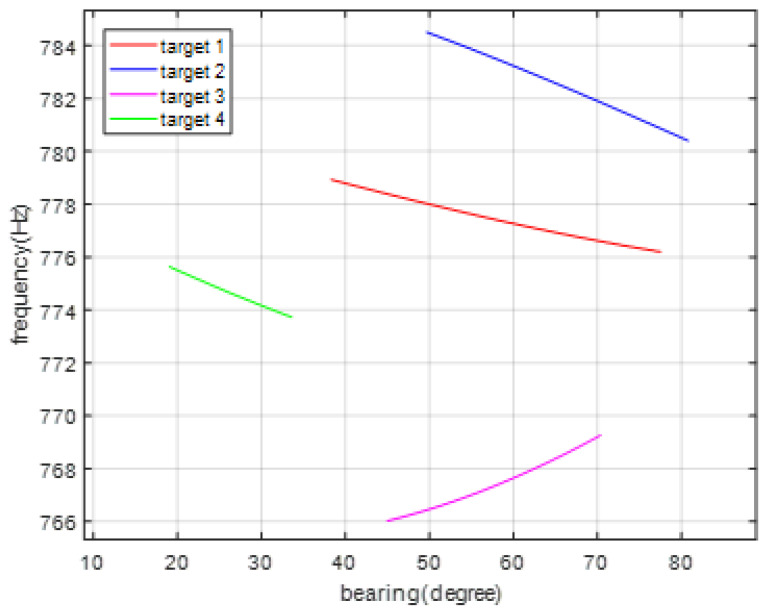
The true measurement of bearing and frequency for the four targets.

The true state for each target is given in Figure 2. There were two crossing moving targets. The sampling period Δt=1 s, the total number of sampling scan is 80. Target 1 arose at the first time step and disappeared at 80 s. Target 2 appeared at 20 s and disappeared at 80 s. Target 3 appeared at 10 s and disappeared at 66 s. Target 4 appeared at 20 s and disappeared at 75 s. The true measurement of bearing and frequency for the four targets is shown in Figure 3.

The standard deviation of the system process noise was 5 m/s^2^. The standard deviation values of the measurement noise for bearing and frequency were $0.5^{\circ }$ and 3 Hz, respectively. The clutter followed a Poisson distribution with clutter intensity $\lambda_{c}=0.3$ over the region [−π/2,π/2]rad×[750,795]Hz—that is, the average clutter each time was 42. For simplicity, the detection probability of all targets was assumed to be the same, and was set to $p_{D,t}=0.98$. We assumed that all targets’ survival probabilities were the same and they were set to $p_{S,t}=0.99$. To inspect the tracking performance of the EKF-based CPHD recursion, the 1000 Monte Carlo process was calculated.

**Figure 4:**
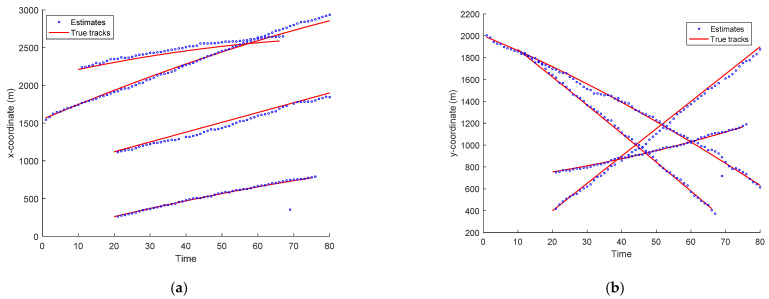
Scans of the target trajectory estimates and true target positions in x- and y-coordinates for CPHD recursion versus time. (**a**) x coordinate, (**b**) y coordinate.

**Figure 5:**
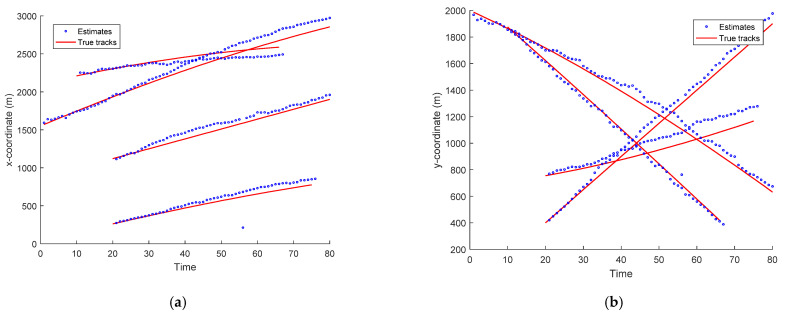
Scans of the target trajectory estimates and true target positions in x- and y-coordinates for the PHD recursion versus time. (**a**) x coordinate, (**b**) y coordinate.

The scans of the targets’ estimated trajectories and true targets’ tracks in x- and y-coordinates versus time for the EKF-based CPHD recursions and PHD recursion are depicted in Figure 4 and Figure 5, respectively. As seen in Figure 4 and Figure 5, the estimated tracks in x- and y-coordinates are similar to the true targets’ tracks for the four targets. This means that the CPHD recursion performed well in tracking multiple targets using the bearing and Doppler measurements. Correspondingly, the PHD algorithm’s tracking accuracy was inferior to that of the CPHD. The calculation time for the EKF-based CPHD for the four targets was 3.46 s per sample run over 80 time scans, implemented in MATLAB R2016a, Intel Core i7 CPU, 16 GB.

**Figure 6:**
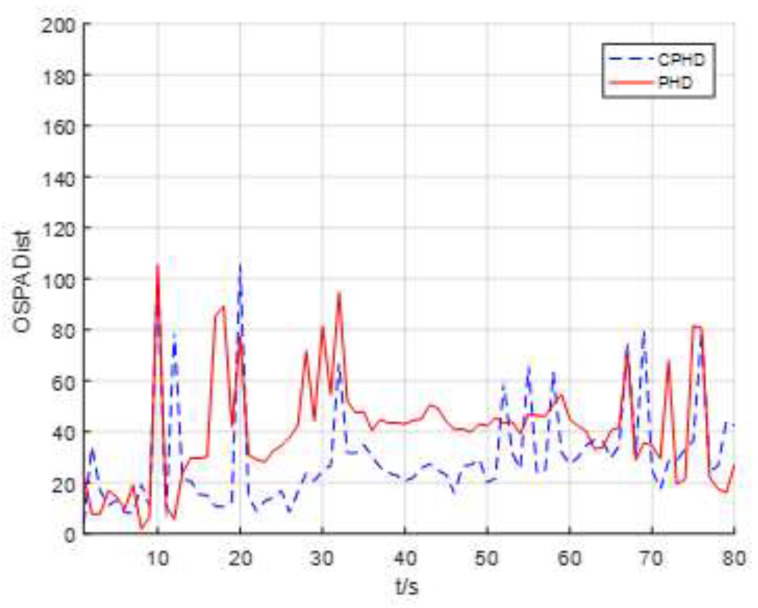
Scans of the average OSPA distance for four targets versus time for both EKF-based CPHD recursion and PHD recursion.

The scans of the average OSPA distance for the four targets versus time for both the EKF-based CPHD recursion and PHD recursion are shown in Figure 6. It appears that the average OSPA distance for the CPHD and the PHD were approximately 30 m and 40 m, respectively. The results also show that the OSPA distance was large when the number of targets was changing, which is normal and means that the CPHD recursion can adapt to changes in targets’ cardinality. It can also be noted that the EKF-based CPHD recursion had a faster reaction to changes in the targets’ cardinality distribution. 

**Figure 7:**
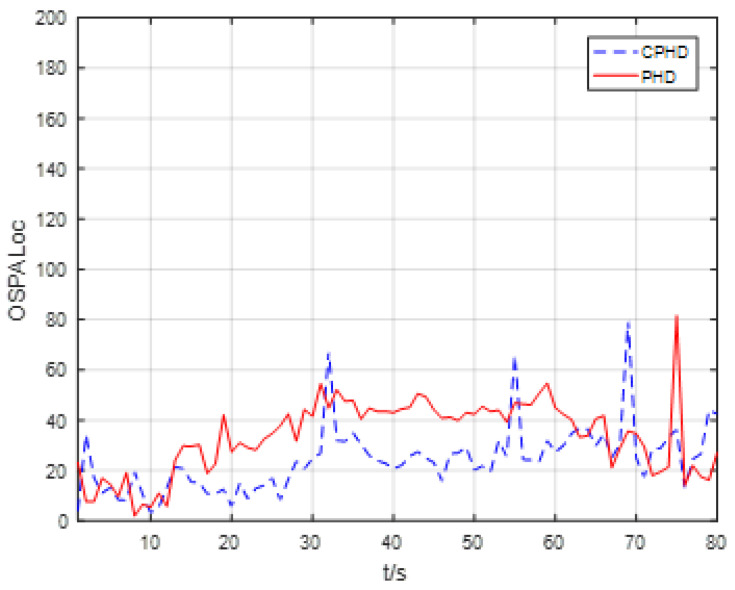
Scans of the average OSPA localization for four targets versus time for the EKF-based CPHD and PHD recursion.

The scans of the average OSPA localization for all four targets versus time for the EKF-based CPHD recursion and PHD recursion are given in Figure 7. In terms of target localization accuracy, Figure 7 shows that the average OSPA localizations for the EKF-based CPHD recursion and PHD recursion were approximately 25 m and 35 m per target, respectively, which demonstrates that the CPHD had a better tracking accuracy performance than the PHD recursion.

**Figure 8:**
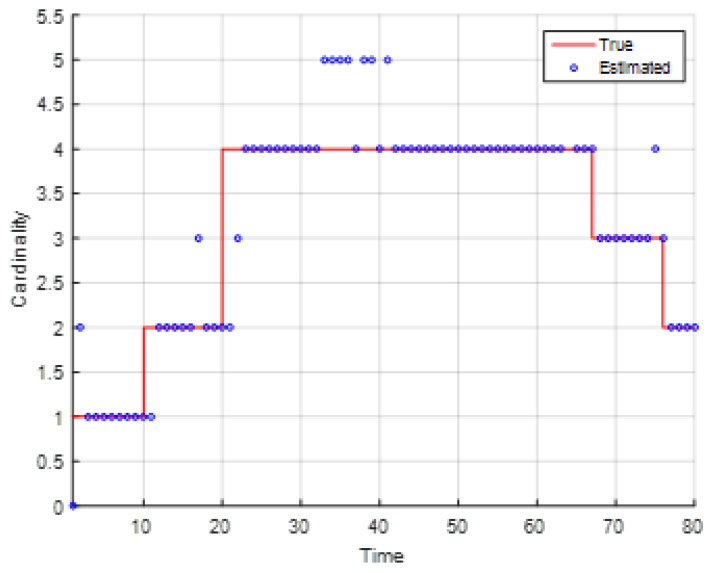
The Monte Carlo average mean of estimated cardinality for the EKF-based CPHD recursion and the true target cardinality.

Figure 8 presents the Monte Carlo means of estimated cardinality for the EKF-based CPHD recursion and the true targets’ cardinality. It can be seen that the EKF-based CPHD recursion could distinguish the appearance of new target and the disappearance of old targets, and it was not likely affected by the incoming bearing and Doppler measurements or clutter. 

## 5. Conclusions

The aim of passive multiple underwater target tracking is to jointly obtain the number of targets in the tracking surveillance space as well as their states (e.g., position, velocity, acceleration) from passive sensor measurements. The greatest merits of passive sonar target tracking are that the passive sonar tracking system is simple and low cost, and can operate covertly due to the use of passive bearing and Doppler measurements. To guarantee that the target range is observable when using the passive measurements, in this paper we employ bearing and Doppler measurements (passive measurements) to track multiple nearly constant turn targets. CPHD is used to handle the data association uncertainty in clutter. The EKF is applied to solve the nonlinearity of bearing and Doppler measurements. The simulation results show that the EKF-based CPHD recursion has accurate propagation in target cardinality due to its fast response, and the estimated tracks in x- and y-coordinates are similar to the true tracks for all targets, indicating that the proposed method has little error. Additionally, the tracking OSPA is small, which means that proposed algorithm has better tracking performance for the passive multiple target tracking problem in a cluttered environment. 

## Data Availability

The data presented in this study are available on request from the corresponding author.
